# What should selective cardiometabolic prevention programmes in European primary care look like? A consensus-based design by the SPIMEU group

**DOI:** 10.1080/13814788.2019.1641195

**Published:** 2019-08-14

**Authors:** Norbert Král, Anne-Karien M. de Waard, François G. Schellevis, Joke C. Korevaar, Christos Lionis, Axel C. Carlsson, Anders Larrabee Sønderlund, Jens Søndergaard, Lars Bruun Larsen, Monika Hollander, Trine Thilsing, Agapi Angelaki, Niek J. de Wit, Bohumil Seifert

**Affiliations:** a Institute of General Practice, First Faculty of Medicine, Charles University, Prague, Czech Republic;; b Department of General Practice, Julius Center for Health Sciences and Primary Care, University Medical Center Utrecht, Utrecht, the Netherlands;; c Nivel (Netherlands Institute for Health Services Research), Utrecht, the Netherlands;; d Department of General Practice & Elderly Care Medicine, Amsterdam Public Health Research Institute, Amsterdam University Medical Centers, Amsterdam, the Netherlands;; e Clinic of Social and Family Medicine, School of Medicine, University of Crete, Greece;; f Division of Family Medicine and Primary Care, Department of Neurobiology, Care Sciences and Society (NVS), Karolinska Institute, Stockholm, Sweden;; g Department of Medical Sciences, Cardiovascular Epidemiology, Uppsala University, Uppsala, Sweden;; h Research unit of General Practice, Department of Public Health, University of Southern DenmarkOdense, Denmark

**Keywords:** Selective prevention, cardiometabolic disease, primary care, general practice, consensus development

## Abstract

**Background:** Selective prevention of cardiometabolic diseases (CMD)—that is, preventive measures specifically targeting the high-risk population—may represent the most effective approach for mitigating rising CMD rates.

**Objectives:** To develop a universal concept of selective CMD prevention that can guide implementation within European primary care.

**Methods:** Initially, 32 statements covering different aspects of selective CMD prevention programmes were identified based on a synthesis of evidence from two systematic literature reviews and surveys conducted within the SPIMEU project. The Rand/UCLA appropriateness method (RAM) was used to find consensus on these statements among an international panel consisting of 14 experts. Before the consensus meeting, statements were rated by the experts in a first round. In the next step, during a face-to-face meeting, experts were provided with the results of the first rating and were then invited to discuss and rescore the statements in a second round.

**Results:** In the outcome of the RAM procedure, 28 of 31 statements were considered appropriate and three were rated uncertain. The panel deleted one statement. Selective CMD prevention was considered an effective approach for preventing CMD and a proactive approach was regarded as more effective compared to case-finding alone. The most efficient method to implement selective CMD prevention systematically in primary care relies on a stepwise approach: initial risk assessment followed by interventions if indicated.

**Conclusion:** The final set of statements represents the key characteristics of selective CMD prevention and can serve as a guide for implementing selective prevention actions in European primary care.

KEY MESSAGES

- The universal concept for CMD prevention in primary care that can guide implementation across Europe is missing.
- EU member states should mandate a programme for selective cardiometabolic prevention, using a stepwise approach, preferably implemented by primary care professionals.

## Introduction

Cardiometabolic disease (cardiovascular disease, diabetes mellitus, and chronic kidney disease; CMD) is a major public health issue worldwide. Apart from being a leading cause of death in most developed countries, CMD often results in significant decreases in quality of life for patients, and considerable increases in associated healthcare costs [1,2]. However, many CMDs are preventable through medical interventions targeting risk factors—such as hypertension and hypercholesterolemia—and lifestyle modifications, like increasing physical activity and quitting smoking [3]. As such, population-based prevention programmes may be able to counter the onset and development of CMD [4], although there is, as yet, no clear consensus about how to develop and implement such programmes.

Several approaches to prevention are described in the literature [5]. Selective prevention, which targets only those at higher than average risk [6], has shown promising results in preliminary research, but evidence from larger studies about its effectiveness is lacking [7]. The debate about the effectiveness of systematic versus opportunistic screening for cardiovascular disease (CVD) and diabetes mellitus is ongoing [8–10]. In addition, there is much debate about the implementation of selective CMD prevention, with questions about optimal setting, preferred strategy, and a logistical approach for identification. The most prominent challenge in selective prevention is how to efficiently identify persons at increased risk in the general population, in order to start the indicated prevention activities [11,12]. Selective CMD prevention has not yet been formally labelled as a key task for general practice [13].

SPIMEU is a cross-European research project, which aims to contribute to the building of greater capacity in relation to the prevention of cardiometabolic diseases in the EU by establishing the feasibility of an innovative approach to identify those at high risk for cardiometabolic diseases (http://www.spimeu.org).

The process and outcome of the expert based consensus procedure that we conducted to identify key characteristics of selective CMD prevention is reported here, to develop a universal concept for selective CMD prevention in primary care that can guide implementation across Europe.

## Methods

### Study design

To develop this concept, a set of statements were developed describing various aspects of the process of selective CMD prevention. These statements were then used as input in a consensus procedure by an international expert panel, using the Rand/UCLA appropriateness methodology (RAM) [14]. RAM comprises an individual, first-round rating of a series of statements that explore the subject of interest by experts. Next, the experts engage in a facilitated group discussion about each statement and finally, these experts participate in a second round of rating, this time with the knowledge added from the group discussion. The result was a set of consensus statements describing the key characteristics of selective CMD prevention.

### Selection of study subjects

The SPIMEU project team (the authors of this article) formulated a set of statements based on the literature, including synthesis of evidence from two literature reviews on barriers to, and facilitators of, selective cardiometabolic prevention among professionals and patients [15,15], and the results of surveys among experts, health professionals and patients about attitudes and practices of selective CMD prevention in the EU [16]. In the next step, following the methodology, the expert panel was set up. The expert panel comprised seven academic general practitioners from five EU countries with experience in group consensus methodology (representatives of the SPIMEU project team), and seven internationally recognized professionals with specific expertise in CMD prevention in Europe (epidemiologists, cardiologists, and other researchers from outside of the SPIMEU project team).

### Measurements and qualitative methods

Statements were mailed to the experts at the end of January 2017, along with background information including a list of references relevant to selective CMD prevention, a summary of the literature reviews, and the survey results. Experts were invited to rate each statement on a 9-point Likert scale (1: completely disagree to 9: completely agree) and were advised to base their ratings on evidence, rather than on personal opinion, and not to emphasize the local/country perspective [17].

The expert panel met for two days in March 2017, to discuss the statements, one by one, including the results of the first-round rating. Literature resources were available online and the discussion was audio-recorded. In situations where it became apparent that the formulation of a statement was suboptimal for panel judgement, the experts reformulated it in consensus. The discussion concluded with a confidential re-rating.

### Outcomes and analysis

The results of the second rating were analysed: the level of agreement was evaluated for each statement and summarized. The statement was considered appropriate when the median rating was between seven and nine, and no rating was in the 1–3 point range. Uncertainty was reported when the median rating was between four and six points, or for any median with three ratings in the 1–3 score range and three ratings in the 7–9 score range [14]. The expert panel authorized the final version of the statements at the end of the two-day meeting.

## Results

The 32 statements that were drafted by the SPIMEU project team covered all key aspects of selective CMD prevention, divided into four domains: (1) background; (2) organization and funding; (3) target group, and methods of identification of risk groups; and (4) provision of selective CMD prevention. Fourteen experts participated in the first rating round, and 12 of them (86%) participated in the consensus meeting and in the second rating round. In the first round, 12 (38%) out of 32 statements were found appropriate, while 20 statements (62%) were rated as uncertain. During the meeting, the participants reformulated a number of statements, some for linguistic and grammar reasons, others to increase their appropriateness. Statement 26 was deleted, based on the panel decision that it duplicated Statement 20. After the second round of rating, 28 (90%) out of 31 statements were agreed upon as being appropriate and three (10%) were considered uncertain. The results are given in relation to the context in the discussion section.

## Discussion

### Main findings

We developed a concept for a selective CMD prevention programme, rooted in 31 statements based on scientific literature which were adopted through a systematic consensus procedure. Selective CMD prevention was considered an effective approach for preventing CMD, and a proactive approach was regarded as more effective compared to case-finding alone.

The most efficient method to implement selective CMD prevention systematically in primary care relies on a stepwise approach: initial risk assessment followed by interventions, if indicated.

Here we highlight the exchange of arguments for the most debated statements.

### Background of selective CMD prevention

Statement 3. Although most incident cases of CMD occur in moderate and low-risk individuals, general health checks offered to the entire population do not reduce all-cause or cardiovascular morbidity or mortality [8] (Table 1). Thus, targeting high-risk individuals rather than mass population screening is the preferred route [18]. Conversely, population-based risk assessment was found to be cost-effective when compared with no screening [19]. There has been diversity in the provision of prevention programmes for CMD across Europe. In some, (e.g. the UK, the Czech Republic) organized programmes have been established and people are actively invited for prevention [20]. In other countries, a case-finding approach is used in general practice (e.g. Denmark). There is limited evidence that a proactive approach (Statement 4) is effective in CMD prevention [6,9,21,22].

Statement 5 provoked a lot of discussion and was ultimately rated as uncertain. CMD and various cancers have a number of risk factors in common. Some conditions increase the risk of others, e.g. a CMD risk is associated with the risk of colorectal cancer [23,24]. A strong patient preference for combining programmes was detected in the SPIMEU patient surveys [15].

The stepwise approach (Statement 6) was suggested in accordance with the Dutch guidelines on preventive consultation, cardiometabolic risk module [6]. This stepwise approach includes an initial risk assessment to preselect people at risk of CMD (e.g. by a self-reported questionnaire), and a subsequent consultation with a general practitioner to complete the risk profile, and to propose tailored preventive interventions, if indicated [5].

### Organization and funding

The understanding of the word ‘to mandate’ in Statement 11 is to give authority to healthcare organizers, health payers, scientific and professional organizations, and healthcare providers to act in order to develop, fund, and implement selective CMD prevention programmes (Table 2).

There was clear agreement on the appropriateness of Statement 13. Nevertheless, the impact of European guidelines—such as joint ESC Guidelines [25], on national guidelines for local adaptation was emphasized.

With regard to Statement 14, and taking into account European diversity, the panel unanimously perceived primary care as the ‘setting,’ which does not always mean general practitioners [6,26].

### Target group and methods of identification of risk groups

Statement 22. Prevention of CMD is suggested to be important at any age (Table 3). The negative cardiometabolic risk profile is shifting towards younger ages. Therefore, it seems appropriate to move the threshold to a lower age. The WHO suggests CMD prevention at age 35–65 in men, and 45–75 in women. The ESC guidelines do not recommend systematic cardiovascular risk assessment in adults under 40 years of age with no known CV risk factor, due to low cost-effectiveness [27]. The Dutch guidelines recommend the use the questionnaire for the preventive consultation from the age 45 to 70 [6]. The SCORE risk assessment is applicable for those over age 40.

A lot of discussion points were raised with Statements 23 and 24, the appropriateness of which were finally rated uncertain. There are differences in the magnitude of the effects of different risk factors between sexes, but the question is if we have enough gender-specific data to produce reliable tools individualized for men and women. Neither the ESC guidelines nor the Dutch guidelines on cardiovascular cardiometabolic prevention suggest gender-specific interventions.

### Provision of selective CMD prevention

Statements 27 and 28 refer to national/regional courses on selective CMD prevention for primary care professionals (Table 4). The way of organizing, certifying, and accrediting these courses should be country specific.

### Strengths and limitations

We believe that the composition of the expert panel with regard to professional background and country resulted in an adequate representation of expertise. The international composition of the group extends the validity of the statements to cultures and languages other than English. The most important limitation of the study is related to the subjective nature of the panel opinions, and the selection of panel members may, therefore, have influenced the outcomes. Other methods could have been used to search for a consensus, but we consider the RAM to be the most suitable tool for combining the best available scientific evidence with the collective judgement of a panel of experts.

The strength of the RAM method is the structured and detailed discussion, though some strong personal opinions, based on country experience, may prevail. The discussion on generic aspects of CMD prevention might have been influenced by differences in healthcare systems, and particularly by their current focus on prevention. The RAM method is a well-established technique for synthesizing group judgements [14]. We produced and explored rather a low number of statements, compared to hundreds or even thousands in other studies. We finally agreed on the appropriateness of a high proportion of statements in comparison to other studies that used the RAM procedure. This might be due to the reformulation of some statements during discussion, but more probably, it reflects the consensus on a need for actions to be taken, in Europe, towards CMD prevention [27,27]. While aiming to prepare universal statements, we could not go into details of some aspects of CMD prevention.

### Relation to other research

The organization of primary care—and its involvement in prevention—differs between countries [28,29]. Authorized guidelines for CMD prevention are available, but there is no generic construct of selective CMD prevention that can be locally adopted and implemented within the system of European primary care. Some key factors in the success of preventive programmes are: compliance within the target group; the support of professionals and healthcare authorities; adequate logistics and funding; and, optimal embedding in regular health services [12].

## Conclusion

The results of this study provide a generic fundament for the design of a stepwise model of selective CMD prevention, which should be further elaborated, through tailored designs, for implementation in general practice in EU member states.

The sustainability of this guide should be regularly reviewed leading to a revision of current knowledge.

## Supplementary Material

SPIMEU Panel Members

## Figures and Tables

**Table 1.** Background of selective CMD prevention.

No.	Statement	1. Rating	Reformulated statements	2. Rating	Median	Range (9-point Likert scale)
1.	Prevention of cardiometabolic disease is one of the most appropriate actions against this major health problem.	Accepted	Yes	Accepted	9	9
2.	Effective interventions on a population level include the creation of a healthier and affordable environment (e.g. displaying food in supermarkets) and actions targeted at promoting a healthy lifestyle (e.g. using stairs instead of elevators).	Accepted	Yes	Accepted	9	6–9
3.	Selective cardiometabolic prevention targets those defined at high risk based on individual risk profile and represents an effective approach for preventing cardiometabolic diseases.	Uncertain	Yes	Accepted	8.5	5–9
4.	Identifying and treating high-risk individuals using a proactive approach is more effective than case-finding alone using the whole population approach.	Accepted	Yes	Accepted	9	6–9
5.	Selective cardiometabolic prevention should be a separate prevention programme not combined with other programmes (e.g., cancer prevention).	Uncertain	No	Uncertain	5.5	3–9
6.	The most efficient method to perform selective cardiometabolic prevention in primary care in a systematic way is through adoption of the stepwise approach: using initial risk assessment followed by interventions if indicated.	Uncertain	Yes	Accepted	9	6–9
7.	The generic step-wise approach for selective cardiometabolic prevention should be adapted nationally/regionally with respect to local conditions into national practical guidelines.	Accepted	No	Accepted	9	7–9
8.	In programmes on selective cardiometabolic prevention, positive effect of worries of patients far outweighs the negative effects.	Uncertain	Yes	Accepted	8	7–9

**Table 2.** Organization and funding.

No.	Statement	1. Rating	Reformulated statements	2. Rating	Median	Range (9-point Likert scale)
9.	EU member states should have programmes that focus on selective cardiometabolic prevention.	Uncertain	Yes	Accepted	9	7–9
10.	Programmes on selective cardiometabolic prevention should be mandated on a national level.	Uncertain	Yes	Accepted	9	7–9
11.	Governments should be responsible for the implementation of policy on selective cardiometabolic prevention.	Uncertain	Yes	Accepted	9	7–9
12.	Costs for selective cardiometabolic prevention should be allocated and protected in regular healthcare financing.	Uncertain	Yes	Accepted	9	8–9
13.	Professional and scientific organizations in each EU country should be responsible for the development of the clinical practice guidelines on selective cardiometabolic prevention.	Accepted	Yes	Accepted	8	7–9
14.	Selective cardiometabolic prevention should preferably be coordinated by primary care.	Uncertain	Yes	Accepted	8	7–9
15.	The effectiveness of a selective prevention programme for cardiometabolic diseases heavily depends on the participation of the target group and their long-term adherence to interventions.	Accepted	No	Accepted	9	7–9
16.	Selective cardiometabolic prevention programmes should first be implemented as a pilot in each respective country and then tailored to the specific contexts that apply to that country.	Uncertain	Yes	Accepted	9	8–9
17.	The data on selective cardiometabolic prevention should be collected, to monitor and scientifically evaluate the programme and allow for adjustments.	Accepted	Yes	Accepted	9	7–9

**Table 3.** Target group and methods of identification of risk group for selective cardiometabolic prevention.

No.	Statement	1. Rating	Reformulated statements	2. Rating	Median	Range (9-point Likert scale)
18.	In order to efficiently identify individuals at high risk of developing cardiometabolic diseases, reliable and relevant data on individuals is required.	Accepted	Yes	Accepted	8.5	5–9
19.	Patients treated for hypertension, diabetes mellitus, cardiovascular disease, chronic renal damage and/or hypercholesterolemia are by definition not a target group for selective cardiometabolic prevention.	Uncertain	Yes	Accepted	9	5–9
20.	The programme should include a validated risk assessment tool for cardiometabolic diseases (CV disease, diabetes mellitus, chronic renal failure).	Uncertain	Yes	Accepted	9	7–9
21.	For the initial approach to patients within selective preventive cardiometabolic diseases programmes optimal local options should be used (post, email, call, internet, direct provision at practices).	Uncertain	Yes	Accepted	8	8- 9
22.	The target population for selective cardiometabolic prevention should at least include individuals aged 40–70 years old.	Uncertain	Yes	Accepted	8	5–9
23.	Men and women should have the same risk assessment tool in selective cardiometabolic prevention.	Uncertain	No	Uncertain	5	1–9
24.	Men and women should have the same intervention in selective cardiometabolic prevention.	Uncertain	No	Uncertain	5.5	1–9
25.	Risk score measures should be validated by each country based on national statistics of cardiometabolic diseases (if available).	Uncertain	Yes	Accepted	9	7–9
26.	Preventive interventions should be based on a complete risk profile.	Uncertain	No	Deleted		

**Table 4.** Provision of selective CMD prevention in primary care.

No.	Statement	1. Rating	Reformulated statements	2. Rating	Median	Range (9-point Likert scale)
27.	During a consultation in primary care an individualized intervention plan should be initiated based on the individual patient’s risk profile.	Uncertain	Yes	Accepted	9	7–9
28.	Each country should have training courses for primary care teams to deliver selective cardiometabolic prevention.	Accepted	Yes	Accepted	9	7–9
29.	Certification and accreditation of this course could facilitate implementation of cardiometabolic prevention.	Uncertain	Yes	Accepted	8	5–9
30.	Selective cardiometabolic prevention tasks should be performed by trained multidisciplinary teams in primary care.	Accepted	Yes	Accepted	9	5–9
31.	For a successful implementation of sustainable nationwide selective prevention of cardiometabolic diseases support from all relevant stakeholders (i.e. national and local government, professional organizations, healthcare insurance companies and patients organizations), is essential.	Accepted	Yes	Accepted	9	7–9
32.	All data on selective cardiometabolic prevention should be recorded in primary care in a structured and validated way to be available for later evaluation.	Accepted	No	Accepted	9	8–9
