# Supplementary material for: What should selective cardiometabolic prevention programmes in European primary care look like? A consensus-based design by the SPIMEU group
Source: Eur J Gen Pract. 2019 Aug 14;25(3):101–8. doi: 10.1080/13814788.2019.1641195 (PMC6713135; doi:10.1080/13814788.2019.1641195)
Supplement: SPIMEU Panel Members [file IGEN_A_1641195_SM9175.pdf]

SPIMEU PANEL MEMBERS

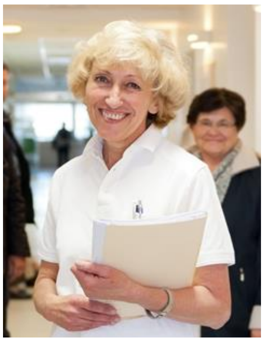

MATEA BULC, ASSOC. PROF., MD, PH.D.

GENERAL PRACTITIONER, TEACHER, RESEARCHER

Department of General Practice University of Ljubljana, Slovinia
General practitioner in Ljubljana Community Health Center
Member of Executive Board of Society of Slovene family physicians from 2006 to 2013
Member of CINDI Slovenia since1990
President of EUROPREV- European Network for Prevention & Health Promotion in Family Medicine and General Practice (since 2010)
She was involved in many research projects with WONCA and WHO in the field of cardio-metabolic prevention.

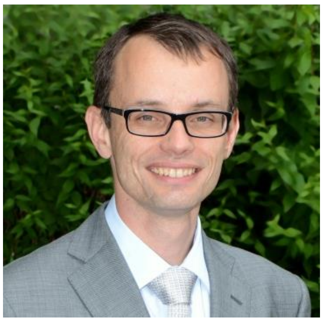

MICHAL VRABLIK, ASSOC. PROF., MD, PH.D.

CARDIOLOGIST, RESEARCHER

Internal Medicine at the 1st Faculty of Medicine, Charles University in Prague, the Czech Republic
Chairman of the Czech Atherosclerosis Society
Member of the Executive Committee of the European Atherosclerosis Society
Member of the board of the Working group of preventive cardiology of the Czech Society of Cardiology.
He participates in the FH screening MedPed program being a leader of the project's national center. He has served as a principal investigator and investigator in more than thirty research projects on familial dyslipidemias and cardiovascular disease risk and has participated in a number of international clinical trials.

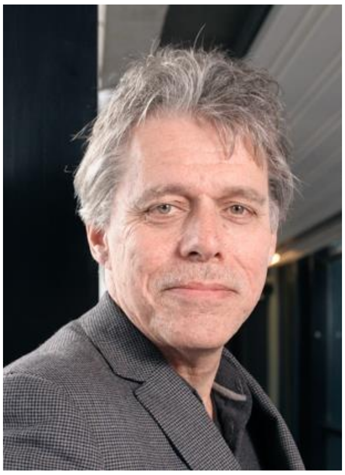

ARNO W. HOES, PROF., MD, PH.D.

EPIDEMIOLOGIST, RESEARCHER

Julius Center for Health Sciences and Primary Care of the University Medical Center in Utrecht, The Netherlands

Professor of Clinical Epidemiology and General Practice

Member of Board of the European Association of Preventive Cardiology and the Heart Failure Association.

His research topics include the prevention of cardiovascular disease and the early diagnosis, of cardiovascular disease. He co-chaired the current Cardiovascular prevention guidelines and is involved in many other ESC activities, including He has (co-)authored over 500 papers in peer-reviewed journals and more than 50 PhD students completed their PhD thesis under his supervision.

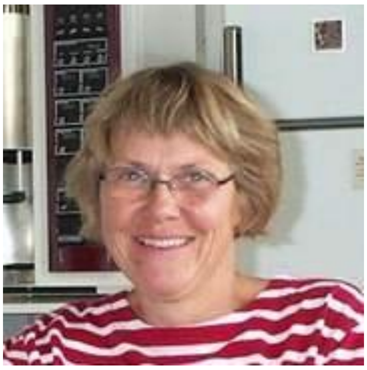

MARGARETA NORBERG, MD, PH.D.

RESEARCHER, PUBLIC HEALTH SPECIALIST

Department of Public Health and Clinical Medicine, Umea University, Sweden

Researcher at Centre for Demographic and Aging Research at Umeå University, Sweden

She has been involved in many studies in the field of cardio-metabolic prevention.

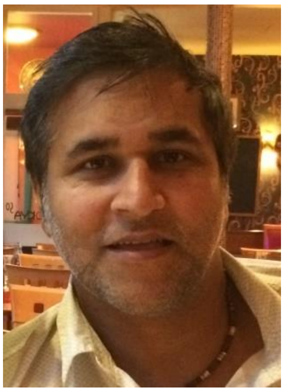

HANIF ISMAIL, PH.D.

RESEARCH FELLOW

Division of Research, Bradford University, UK

An expert reviewer for the UK National Institute for Health Research (NIHR) programme grants and several academic journals.

A mixed methods social scientist with an interest in health inequalities, ethnicity, chronic disease management, early interventions and behaviour change. He has 17 years' experience in conducting mixed methods health research within Universities and both Primary (GP practices)/ Secondary (hospitals) care settings.

At the moment he is working on a multi-million pound study that focuses on Improving the Safety and Continuity of Medicines Management at Care Transitions for patients with Heart Failure.

In terms of qualifications he has a PhD in Health Sciences from the University of York, a Masters degree in Social policy and a BSc in 'Sociology/Social Psychology', both obtained at the University of Bradford UK.

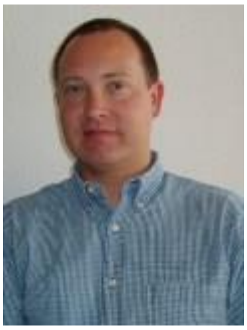

JANUS LAUST THOMSEN, PROF, PH.D.

GENERAL PRACTITIONER, RESEARCHER

The Faculty of Medicine, Department of Clinical Medicine Aalborg University, Denmark

Department of Public Health, University of Aarhus.

He has been involved in many research studies and opublicvcation in the field of cardiovascular and cancer prevention.

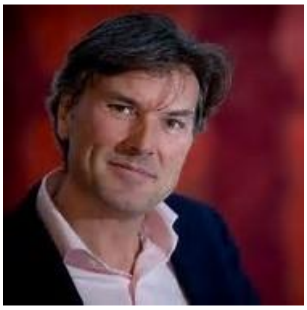

RODERIK KRAAIJENHAGEN, MD, PH.D.

CARDIOLOGIST, ORGANIZATOR

Medical director of Institute for Prevention and E-Health Development (NIPED), knowledge institute for integrated prevention and eHealth, The Netherlands

Developer and designer of the PreventionCompass and Personal Health Check with underlying scientific knowledge- and decision support system for integrated risk profiling and personalized follow-up.

Founder and Chairman of Hearts4People foundation; a healthcare provider for specialized cardiometabolic care in an 'out of hospital' setting.

Founder and director of Hearts4People Inc.; a corporation specialized in eCaoching and eHealth and owner of the 'HealthePortal' and 'Mijn HealthePortal' (health management platform specialized in guided self-management and lifestyle support).

Chairman of 'Begeleide Zelfzorg' (guided self-management) foundation; a national thrombosis service and expert center providing an integrated thrombosis management approach (TromboVitaal) with checkpoints at pharmacists and general practitioners, facilitating self- management and providing 24/7 eHealth support by thrombosis specialists.

Founder and director of CardioVitaal Inc.; a healthcare provider for multidisciplinary cardiac rehabilitation focusing on self-regulation and self-management using eCoaching and telemonitoring.

Founder and director of the Arterium in Amsterdam: a medical clinic specialized in cardiometabolic disease and cardiac rehabilitation.

National Coordinator for cardiovascular disease prevention of the European Society of Cardiology

Chairman of the Committee for Cardiovascular Prevention and Rehabilitation of the Dutch Cardiology Society (from 2010 to May 2016)

Chairman of the National Multidisciplinary Council for Cardiac Rehabilitation (LMDOH) Member of various guideline committees, such as 'cardiovascular risk management', 'smoking cessation', 'hypertension' and 'cardiac rehabilitation'.

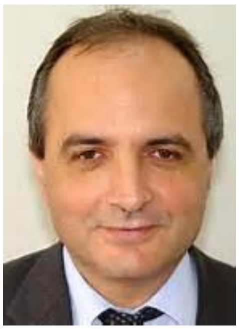

PROF. CHRISTOS LIONIS, MD, PHD, HON FRCGP

ACADEMIC, RESEARCHER

Professor of General Practice and Primary Health Care, and Director of the Clinic of Social and Family Medicine within the School of Medicine at the University of Crete.

Prof Lionis has extensive experience in implementing educational and research initiatives within PHC with over 250 publications cited in PubMed. Prof. Lionis is actively involved in the development of Primary Care and General Practice in Greece and leads a productive research group which is focused on multidisciplinary approaches to health care, and consideration of social and psychological factors affecting health.

Prof. Lionis has led multiple large scale collaborative research programs within Greece and the European Union (e.g. FP7 projects: OTC SOCIOMED, TRANSFORM, RESTORE, and EUWISE).

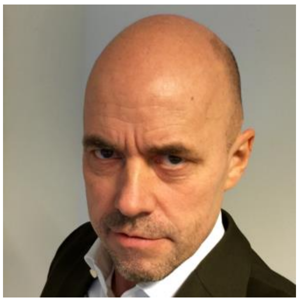

JENS SØNDERGAARD PROF., GP, CLIN. PHARM. MD PHD ,

ACADEMIC, RESEARCHER

The Director of the Research Unit of General Practice at The University of Southern Denmark. He is the leader of a number of research groups, he has been chair of the Research Board of the Danish College of General Practitioners (2003 –2010), and chair of a number of national committees and member of a number of international committees.

He has extensive research experience with main focus on Clinical research and public health research on chronic obstructive lung disease and comorbidities. He has authored or co-authored approximately 110 publications in peer-reviewed indexed journals, more than 100 other publications (clinical guidelines, reports, letters, interviews and chapters), more than 110 oral presentations and more than 70 posters at scientific conferences and more than 130 abstracts. He has been a part-time GP since 1997 and holds a specialist degree in clinical pharmacology.

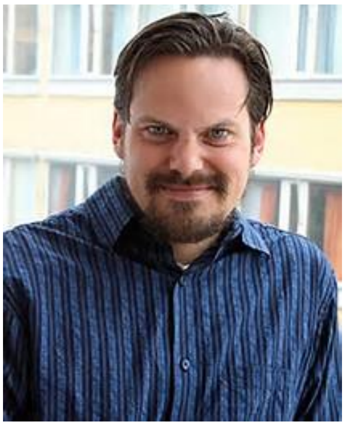

AXEL C. CARLSSON, DR., MSC, PHD

CLINICAL PHARMACIST AND TEACHER

Clinical pharmacist and a teacher in epidemiology, responsible for the Research School in Family Medicine at Karolinska Institutet. After finishing his PhD on the epidemiology of hypertension in 2009, Axel has done research on everything from lifestyle advice and obesity to cardiometabolic and cardiorenal diseases, and their complications.

He has authored or co-authored 47 publications in peer-reviewed indexed journals, among them in the top-ranked journals for obesity, nephrology and hypertension.

Axel has also acted as a scientific co-author of the National Diabetes Guidelines, and the updating of these guidelines for the National Board of Health and Welfare in Sweden.

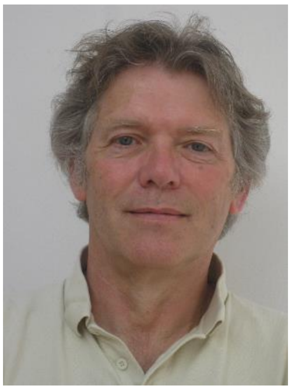

PROF. FRANÇOIS SCHELLEVIS, MD, PH.D

GENERAL PRACTITIONER, EPIDEMIOLOGIST

Coordinator of the SPIM EU project. He has been trained and has worked as a part-time general practitioner during 15 years, and as epidemiologist. He is employed by NIVEL since 1997 and since 2006 he is a part-time professor of General Practice at the VU University Medical Center in Amsterdam, the Netherlands.

Prof. Schellevis is (co-)author of more than 200 international peer reviewed scientific publications.

He has coordinated several EC funded projects within previous SanCo Health programmes ("Health Monitoring" and "Health indicators from Primary Care") and the FP7 programme (e.g. the "APRES study").

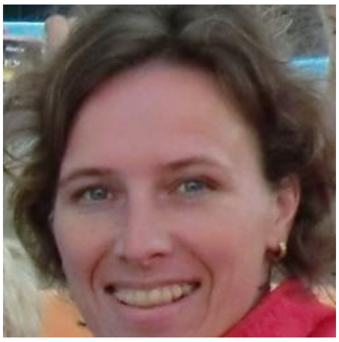

MONIKA HOLLANDER, MD, PH.D.

ASSIST. PROF. , RESEARCHER

Julius Center for Health Sciences and Primary Care of the University Medical Center in Utrecht, The Netherlands.

Representing one of the partners in The SPIMEU project. Cardio-metabolic prevention has been her research interest for many years.

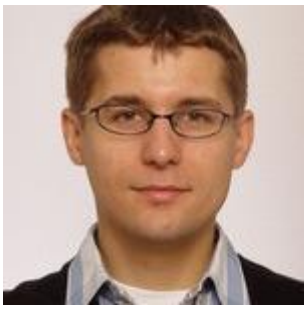

NORBET KRAL, MD

GENERAL PRACTITIONER, RESEARCHER

Department of General Practice, 1st Faculty of Medicine, Charles University

Representing one of the partners in The SPIMEU project. He has been involved in studies and publications on cancer and cardio-metabolic prevention.

MODERATORS:

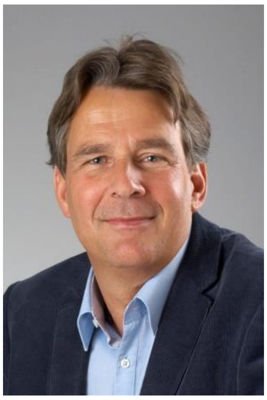

NIEK J DE WIT, PROF., MD PHD FRCPE

GENERAL PRACTITIONER, RESEARCHER

Julius Center for Health Sciences and Primary Care of the University Medical Center in Utrecht, The Netherlands. He is representing one of partners in the SPIMEU project.

He has been practising as general practitioner for more than 20 years. In 2009 he was appointed as professor in Primary Care and head of department at the Julius Center of Health Sciences and Primary Care of the University Medical Center in Utrecht. He is also medical director of the Julius Health Centers in Leidsche Rijn, and member of the advisory board of the National Quality Institute of the Dutch Ministry of Health. In his research he focusses on the development and evaluation of health care innovations in primary care practice. He is co-author of more than 150 peer reviewed publications.

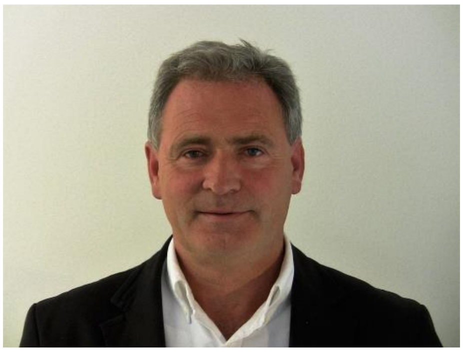

BOHUMIL SEIFERT, ASSOC. PROF., MD, PH.D.

GENERAL PRACTITIONER, RESEARCHER

He is representing one of partners in the SPIMEU project. He is general practitioner and head of the Department of General Practice, 1st Faculty of Medicine, Charles University Prague. He is also the Scientific Secretary of the Czech Society of General Practice. He completed his doctoral studies in preventive medicine in 2007. Bohumil Seifert's research interest has been in the epidemiology and management of gastrointestinal disorders in primary care and in secondary prevention of colorectal cancer. He has been a leading person in colorectal cancer screening programme in the Czech Republic since the beginning in 2000. He was involved in research and publications in the field of cancer and cardio-metabolic prevention.
